# Pegylated interferon α enhances recovery of memory T cells in e antigen positive chronic hepatitis B patients

**DOI:** 10.1186/1743-422X-9-274

**Published:** 2012-11-16

**Authors:** Yong Zhe Liu, Feng Qin Hou, Peng Ding, Yuan Yuan Ren, Shi Hong Li, Gui Qiang Wang

**Affiliations:** 1 Department of Infectious Diseases and Research Center for Liver Diseases, Peking University First Hospital, Beijing, 100034, People’s Republic of China; 2 Institution of Public Health Inspection in Ningxia Hui Autonomous Region, Ningxia Hui Autonomous Region, 750004, People’s Republic of China; 3 College of Public Health, Hebei United University, Hebei province, 063000, People's Republic of China

**Keywords:** Chronic hepatitis B, Pegylated interferon-α therapy, Memory T cell, Intracytoplasmic cytokine staining (ICCS)

## Abstract

**Background:**

Interferons (IFNs) are a group of cytokines commonly used in the clinical treatment of chronic hepatitis B (CHB) patients. Their therapeutic effects are highly correlated with recovery of host antiviral immunity. Clearance of hepatitis B virus (HBV) is mediated partially by activated functional memory T cells. The aims of the present study were to investigate memory T cell status in patients with different outcomes following pegylated interferon-α (IFN-α) therapy and to identify new biomarkers for predicting antiviral immune responses.

**Methods:**

Peripheral blood cells were isolated from 23 CHB patients who were treated with pegylated IFN-α at week 0 (baseline) and week 24. Co-expression of programmed death-1 (PD-1) and CD244 in CD45RO positive T cells, as well as a subset of CD127 and CXCR4 positive memory T cells were assessed. In addition, perforin, granzyme B, and interferon-γ (IFN-γ) expressions were also analyzed by flow cytometric analysis after intracytoplasmic cytokine staining (ICCS). Peripheral blood mononuclear cells (PBMC) isolated at week 24 were re-challenged with exogenous HBV core antigen, and the percentage of IFN-γ expression, serum HBV DNA loads, and ALT (alanine aminotransferase) levels were evaluated.

**Results:**

At week 24, PD-1 and CD244 expression in CD8 memory T cells were down-regulated (*P* < 0.05, *P* < 0.05, respectively), along with decreased HBV DNA loads (*P* < 0.05), while the expressions of partial effector molecules in CD8 and CD4 memory T cells was up-regulated (*P* < 0.05,*P* < 0.05, respectively), especially in the responders. CD127 and CXCR4 were highly expressed in CD8 memory T cells after pegylated IFN-α treatment (*P* < 0.05), which was inversely correlated with HBV DNA loads (*r* = −0.47, *P* = 0.001). The responders had a higher IFN-γ expression in memory T cells than the non-responders did after HBV antigen re-stimulation *in vitro*.

**Conclusion:**

Pegylated IFN-α treatment enhanced recovery of memory T cells in CHB patients by down-regulating inhibitory receptors and up-regulating effector molecules. The expressions of CXCR4 and CD127 in CD8 memory T cell may be used as biomarkers for predicting the outcome of treatment.

## Introduction

Chronic hepatitis B virus infection is the most common cause of liver cirrhosis and hepatocellular carcinoma in China [1,2]. The chronicity of HBV infection depends on viral factors, host immunity, and the intrahepatic microenvironment. Adaptive immunity plays a vital role in antiviral effects in the liver and peripheral infections [3,4]. However, during the progression of chronic HBV infection, T cell dysfunction is too profound to effectively control viral replication, leading to a decline of viral clearance. More specifically, exhaustion and depletion of effector cells hinder T cell homeostasis and antigen-specific memory T cell formation [5]. During chronic HBV infection, naive T cells are primed by antigens and then differentiate into effector T cells. Unless the infection is cleared following antigen clearance, and the intrahepatic inflammation is diminished or substantially reduced, partially functional effector T cells can further differentiate into highly polyfunctional memory T cells. Antigen specific memory T cells keep on self-renewing and have the potential to proliferate and differentiate upon encountering antigens again [6,7]. In other words, memory T cells have recall reaction ability. They are capable of producing multiple cytokines, such as IFN-γ, TNF-α, and IL-2, becoming cytolytic, and proliferating vigorously to become activated functional cells [8]. These cells also have high survival capability and are maintained for long periods in the absence of antigen.

Pegylated IFN-α is one of the most common therapeutic cytokines in the treatment of chronic HBV infection [9,10]; it relies on host immunity mobilization to produce antiviral components, such as interleukin-12 and IFN-γ, and to induce the differentiation of adaptive immunocytes, which block viral protein synthesis and assemblage of viral structures. Until now, few studies focused on memory T cell variation and the immunologic consequences of pegylated IFN-α treatment in chronic hepatitis B infection [11]. The goal of this study is to investigate the influence and variation of memory T cell status after pegylated IFN-α treatment by evaluating the expression of inhibitory receptors, effector molecules, and chemokine receptors according to the viral DNA loads [12], and the capacity to release effector cytokines by *in vitro* antigen stimulation [13].

## Results

### Characteristics of patients

To evaluate the effect of pegylated IFN- α treatment on memory T cells in CHB infection, 23 CHB patients were divided into responders (*n* = 9) and non-responders (*n* =14) at week 24. The patients’ characteristics before treatment are summarized in Table 1. The responders were patients with normal ALT and HBV DNA loads that had decreased more than 3log values, and/or e antigen seroconversion after 24 weeks of the treatment; the rest of patients were defined as non-responders.

**Table 1:** Characteristics of the patients

**Characteristics**	**All patients**	**Responders**	**Non-responders**
Cases	23	9	14
Age(year)	36 (23–52)	35 (23–47)	37 (23–52)
Sex(male/female)	15/8	5/4	10/4
HBVDNA loads [log (IU/mL)]	6.85 (4.65-8.06)	6.66 (5.23-7.77)	6.97 (4.65-8.06)
ALT(IU/L)	113.6 (42–285)	125.3(42–251)	79.5 (43–285)
HBVgenotype (B/C)	6/17	2/7	4/10

Data are presented as median (range).

### PD-1 and CD244 expressions were down-regulated in memory T cells

**Figure 1:**
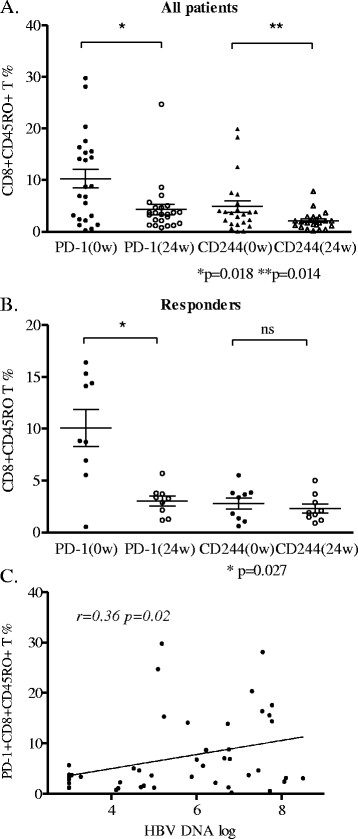
**Percentage of inhibitory receptors expression on CD8 memory T cells in CHB patients.** (**A**) Percentage of PD-1 and CD244 expression on CD8 memory T cells in all patients at week 0 and week 24. (**B**) Percentage of PD-1 and CD244 expression on CD8 memory T cells in responder patients at week 0 and week 24. (**C**) Correlation between PD-1 expression on CD8 memory T cell and HBV viral loads.

PD-1 and CD244 expressions in CD8 memory T cells (CD8 + CD45RO+) were simultaneously down-regulated along with decreased HBV DNA loads after pegylated IFN-α treatment in all patients (*P* = 0.018, *P* = 0.014, respectively), when comparing to baseline expressions (Figure 1A). In addition, the responders had significant loss of PD-1 expression in CD8 memory T cells (*P* = 0.027, Figure 1B), but CD244 expression in CD8 memory T cells did not show the same trend (*P* = 0.57, Figure 1B). Furthermore, the PD-1 expression in CD8 memory T cells and the HBV DNA loads were moderately correlated in all patients (*r* = 0.36, *P* = 0.02, Figure 1C) whereas CD244 expression in the same subset of T cells was not correlated with HBV DNA loads. The expression of PD-1 and CD244 in CD4 memory T cells had neither significant differences nor correlations with DNA loads in all subjects observed (data not shown).

### IFN-γ, perforin and granzyme B expressions were up-regulated in memory T cells

**Figure 2:**
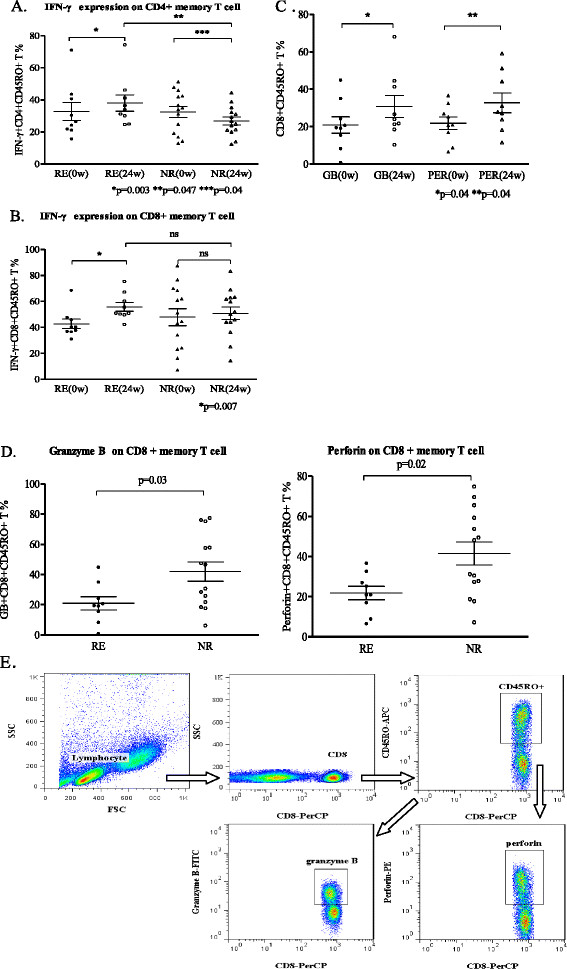
**Distributions of different effector molecules expression on memory T cells.** (**A**) Percentages of IFN-γ expression on CD4 memory T cell between the responders (RE) and the non-responders (NR), which represented the effector molecule of Th1 subsets. (**B**) Percentages of IFN-γ expression on CD8 memory T cells between the responders and the non-responders, which represented the effector molecule of cytotoxic T cells. (**C**) Percentages of granzyme B and perforin expression on CD8 memory T cells comparison in the responders at week 0 and week 24. (**D**) Percentages of granzyme B and perforin expression on CD8 memory T cells between the responders and the non-responders at week 24. (**E**) Gating strategy in analysis of granzyme B and perforin expression on CD8 memory T cells by ICCS.

Upon stimulation with PMA and ionomycin, we tested the release of IFN-γ by memory T cells. The results showed that IFN-γ expression on CD4 + CD45RO + T cells was significantly different between week 0 and week 24 of treatment in both the responders (*P* = 0.003) and the non-responders (*P* = 0.04, Figure 2A). In addition, IFN-γ expression on CD4 memory T cells after the treatment was higher in the responders than that in the non-responders (*P* = 0.047, Figure 2A).

While the expression of IFN-γ on CD8 memory T cells in the responders is significantly different between week 0 and week 24 of treatment (*P* = 0.007, Figure 2B), there was no significant difference in the non-responders between week 0 and week 24 of treatment, nor was there any significant difference between the responders and the non-responders at week 24.

By using gating strategy analysis as shown in Figure 2E, we found that the expressions of granzyme B (*P* = 0.04) and perforin (*P* = 0.03) on CD8 memory T cells at week 24 in the responders were higher than those at week 0 (Figure 2C) but lower than those in the non-responders (*P* = 0.03, *P* = 0.02, respectively, Figure 2D).

### CD127 expression on CD8 memory T cells was up-regulated

**Figure 3:**
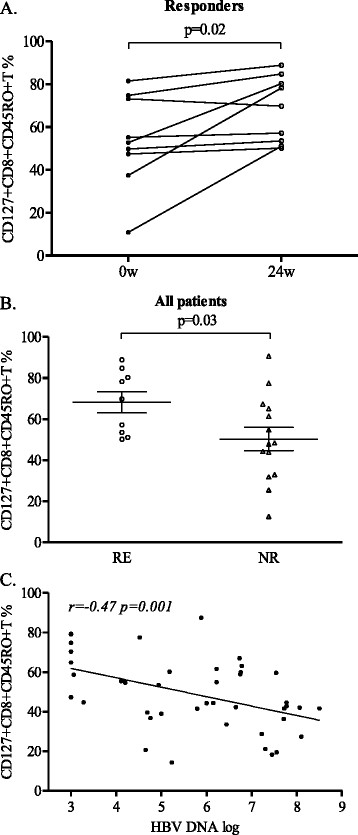
**Percentage of CD127 expression on CD8 memory T cells.** (**A**) Percentage of CD127 expression on CD8 memory T cells comparison between week 0 and week 24 in the responders. (**B**) Percentage of CD127 expression on CD8 memory T cells comparison between the responders (RE) and the non-responders(NR) at 24 week. (**C**.) Correlation between the percentage of CD127 + CD8 memory T cells and HBV loads.

The results from flow cytometric analysis showed that CD127 expression on CD8 memory T cells was up-regulated in the responders after 24 weeks of treatment (*P* = 0.02, Figure 3A). The percentage of CD8 memory T cells that expressed CD127 was higher in the responders than in the non-responders at week 24 (*P* = 0.03, Figure 3B). However, there was no difference in the percent of CD4 memory T cells that expressed CD127 between the responders and the non-responders at week 24 (data not shown). The percentage of CD127 + CD8 + CD45RO + T cells had a negative correlation with HBV viral load (*r* = −0.47, *P* = 0.001, Figure 3C).

### CD8 memory T cells had elevated expression of CXCR4 in responders

**Figure 4:**
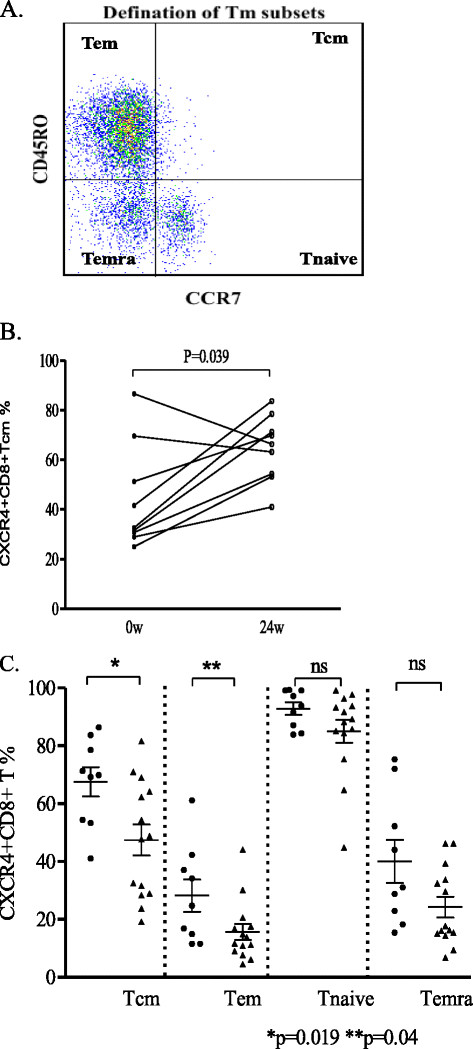
**Distribution of CXCR4 expression on CD8 memory T subsets.** (**A**) Gating strategy on defining memory T cell subsets: Naïve cells were defined as CD45RO-CCR7+ group. Memory cells were defined as CD45RO + CCR7+ group (central memory, Tcm) or CD45RO + CCR7- group (effector memory, Tem). (**B**) Percentage of CXCR4 expression on CD8 Tcm in responders comparison between week 0 and week 24. (**C**) Percentage of CXCR4 expression on CD8 memory T subsets comparison between responders (black dots) and non-responders(black triangle) at week 24.

After defining various subsets of memory T cells (Figure 4A), we found that a small percent of central memory T (Tcm) cells in most CHB patients had no significant difference in percentage in all patients (data not shown) between week 0 and week 24, whereas the CXCR4 expression on CD8 + Tcm cells was up-regulated at week 24 in the responders (*P* = 0.039, Figure 4B). Furthermore, the percentage of CXCR4 expressing CD8 + Tcm and Tem in the responders was higher than that in the non-responders (*P* = 0.019, *P* = 0.04, respectively, Figure 4C). There was no significant difference in the CD8 naive and CD4 memory T cell subsets between the responders and the non-responders.

### IFN-γ release increased upon HBV antigen re-challenge *in vitro*

**Figure 5:**
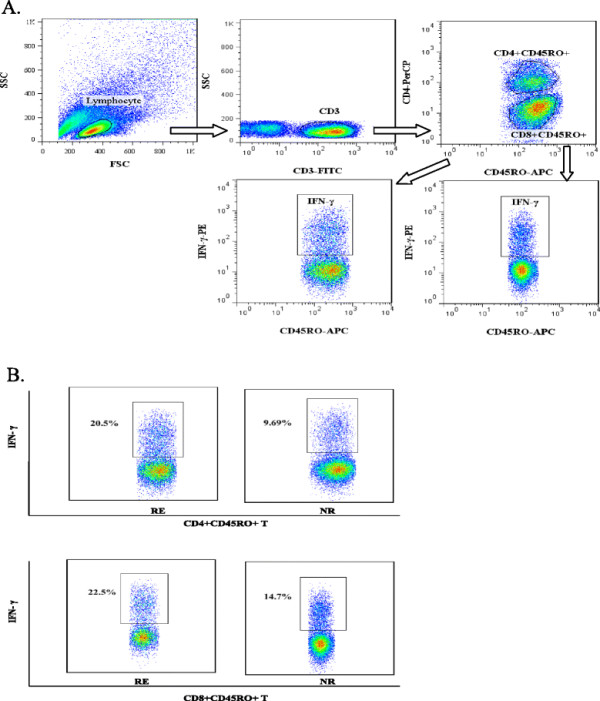
**Percentage of IFN-γ expression on memory T cells after isolated PBMC incubated with exogenous HBV core antigen *in vitro*.** (**A**) Gating strategy in analysis of IFN-γ expression on CD4 and CD8 memory T cells after the stimulation. (**B**) An example of IFN-γ expression on CD4 and CD8 memory T cells each from the responders and the non-responders under the same stimulating condition.

After incubating with HBV core antigen (1 ng/ml) for 72 hours *in vitro*, PBMC from the patients were stimulated with PMA and ionomycin. The expression of IFN-γ was analyzed using flow cytometric analysis after ICCS procedure as shown in Figure 5A. Replicated experiments were performed in each group simultaneously. The percentage of IFN-γ expressing CD4 and CD8 memory T cells were higher in the responder group than those in the non-responder group (Figure 5B), indicating that memory Th1 and CTL in the responders predominate in similar antigen recurrence as well.

## Discussion

Effector T cell dysfunction plays a vital role in the majority of chronic HBV infection and the severity of CHB progression [14,15]. It has been reported that recovery of T cell function might reverse antiviral effect in host immunity and hinder the progression of chronic inflammation and fibrosis [16,17]. However, little is known about the status of memory T cells and the related functional recovery after pegylated IFN- α treatment. During interferon treatment in chronic HBV infection, CD8+ and CD4+ T cell differentiation following virus clearance results in the formation of high quality, long-lived memory cells [18]. Two key features of memory immunocytes are long-term persistence in the absence of antigen and rapid response upon re-exposure to the pathogen, which allow them to transfer to effector cells and confer protective immunity. This recall response is characterized by rapid elaboration of effector functions such as cytotoxicity, cytokine production, effector T cell proliferation accompanied by substantial increase in the number of activated T cells, and effector T cell migration to infection sites [19,20].

In this study, we found that pegylated IFN- α treatment enhanced CD8 memory T cells recovery by down-regulating expression of inhibitory receptors on memory T cells in CHB patients. Our previous study [21] indicated that PD-1 and its ligand—programmed death ligand-1(PD-L1), expressed mainly on antigen present cells (APC)—were down-regulated in intrahepatic and peripheral lymphocytes after pegylated IFN- α treatment. As HBV antigen or viral load increases, these virus specific CD8+ T cells may express high levels of PD-1 in response to polyfunctional cell loss and effector cell dysfunction in a hierarchical manner. The pattern of inhibitory receptor co-expression and the frequency of receptors simultaneously expressed by the same CD8 memory T cell can substantially affect the severity of antiviral effector cell dysfunction [22,23]. CD244 is a novel biomarker, which has been reported to be mainly expressed on nature killer cells, involved in partial exhaustion of activated T cells [24]. Interestingly, PD-1 and CD244 co-expression facilitates the increased susceptibility to apoptosis of memory T cells, and reversing the expression of these receptors implies the recovery of antiviral immunity. In line with our previous study regarding CHB patients treated with interferon-α-2b, which attained down-regulation of inhibitory receptors accompanied with viremia control [25], we found that PD-1 and CD244 were down-regulated preferentially in CD8 memory T cells during pegylated IFN- α treatment. Dynamic down-regulation of inhibitory receptor expression on CD8 memory T cells indicates functional T cell recovery. However, these results were not observed on CD4 memory T cells, which may be due to limited samples and need further investigation.

Chemokines and their receptors are essential in memory T cell migration and homing and directing mature activated T cells to lymphoid tissues and other immune microenvironments that are suitable for their differentiation and function. The chemokine receptor CXCR4 belongs to the superfamily of G-protein-coupled receptors [26,27]. A recent report has highlighted the role of CXCR4 as a prognostic marker in various types of cancer, including leukemia and breast cancer [28]. CXCR4 and CCR5 are also co-receptors for HIV entry into human cells [29], but their roles in viral hepatitis have not yet been addressed. Formation of highly functional memory T cells accompany viral control during antiviral treatment. Once they encounter the viral antigens again, the populations of memory CD4 + and CD8+ T cells expand in lymphoid tissue and then immigrate to the periphery where they bind with their ligands and are then activated to take effect. The chemokine receptors that are highly expressed on memory T cells not only prepare them for life in the periphery but are also correlated with the outcome of antiviral treatment. CCR7+ T cmcells are capable of proliferating and transferring to CCR7- Tem cells after activation, while cytokine production are enriched in Tem cells [30]. Interestingly, we found that CXCR4 was highly expressed on a subset of CD8 memory T cells in the responders, suggesting that memory T activation is accompanied with dramatic alterations in chemokine responsiveness. In our investigation, we found that CXCR4 was more highly expressed on CD8+ Tcm cells of the responders than on those of the non-responders, indicating that migration of Tcm (CD45RO + CCR7+) reverted after pegylated IFN-α treatment. Therefore, we postulate that higher co-expression of CCR7 and CXCR4 on memory T cells represents the potent mobility that can affect targets in antiviral treatment. These memory T cells with high chemokine receptor expression may serve to ensure a robust cycle of antigen-specific memory T-cell activation and proliferation. Unfortunately, CXCR4 and CCR7 tend to shed in culture in response to antigen stimulation, and it is hard to measure the effector cytokines expressed on memory T subset in this scenario.

CD127 is the α chain of the interleukin-7 (IL-7) receptor, and it’s mainly expressed on T cells. IL-7 is a survival cytokine of memory T cells and forms a feedback loop with its receptor expression in the immune milieu. Chronic viral infection induces a significant decrease in CD127 expression on CD8+ T cells [31,32]. Studies elucidated that high CD127 expression indicates the enhanced function of memory T cells in viral clearance. Reported data strongly support that the rejuvenated cellular responses correlate with the homeostasis and proliferation of functional T cells, but the underlying mechanisms remain unclear [33-35]. In this study, we found that CD127 expression on CD8 memory T cells was significantly higher in the responders than in the non-responders, which indicated that proliferation took place predominately in the responders after pegylated IFN-α treatment and reinvigorated the T cells activation. However, the detailed mechanisms and immunological changes need further investigation. Meanwhile, CD127 expression was tightly correlated with HBV DNA load, suggesting that up-regulated CD127 expression in pegylated IFN-α treatment may be a novel biomarker to predict the outcome of antiviral treatment.

T cell dysfunction becomes progressively worse as viral load increases or inhibitory signals are upregulated. Bertoletti et al. indicated that interferon release lagged behind innate immune response in certain HBV infections. CD8 + memory T cells in the adaptive immune system take action upon encountering replicating HBV virus [36,37]. Tcm proliferation and differentiation into effector T cells contribute to pathogen elimination by perforin-dependent cytolysis and secretion of other functional cytokines [38]. In this study, the expression of effector molecules such as perforin and granzyme B on memory T cells were up-regulated, which enhanced the host antiviral activity by using exogenous interferon. But the perforin and granzyme B expressions on memory CD8+ T cells in responders were not higher than those in the non-responders at week 24. We speculate that the effector molecules synthetized in CD8 memory T cells mainly induce apoptosis and necrosis of virally infected cells, whereas memory T cells in the responders acquired viral control via the feedback of lower granzyme B and perforin expressions. IFN-γ expression on both CD4 and CD8 memory T cells was up-regulated after pegylated IFN-α treatment, indicating the potent generation of type 1 T helper cells and cytotoxic activity in the responders. These classical cell types are in charge of antiviral immune responses. IFN-γ expression in memory T cells was up-regulated dramatically after *in vitro* HBV core antigen re-challenging, reflecting the sensitive and potent capability of memory in the responders, which may predict long-term viral control after the treatment.

Taken together, we found that memory T cells recovered after pegylated IFN- α treatment via down-regulation of inhibitory receptors, up-regulation of chemokine and survival cytokine receptors, and enhanced production of effector molecules. Therefore, pegylated IFN- α regulates memory T cell functions during persistent chronic HBV infection. A better understanding of the characteristics and mechanisms responsible for memory T cell dysfunction and recovery during antiviral therapy helps one to develop sensitive immunological markers for predicting the outcome of antiviral treatment and vaccine approaches that reduce the disease burden of intractable chronic infections. These results were obtained from a small scale follow-up of CHB patients treated with pegylated IFN-α. A further study is needed with increased sample size and a longer period of follow-up.

## Conclusion

Pegylated IFN-α treatment enhanced recovery of memory T cells in CHB patients via down-regulating inhibitory receptors PD-1 and CD244 and up-regulating effector molecules perforin, granzyme B and IFN-γ. The responders had a rapid and potent recall response upon reencountering viral antigen *in vitro*. The expression of CXCR4 and CD127 on CD8 memory T cell may be used to predict outcome of the treatment.

## Materials and methods

### Patients and study design

The consecutive CHB patients enrolled in the Peking University First Hospital Research Center for Liver Diseases and Infectious Diseases Department were followed between February 2011 and November 2011. The protocols involved in this study were approved and monitored by the Peking University First Hospital Research Ethics Board, and all patients signed informed consent forms in accordance with the Declaration of Helsinki. Diagnosis of chronic HBV infection was established as previously described [39]. Samples collected from e antigen positive chronic HBV individuals were naïve to nucleotide analogs (NAs) or interferon. All subjects were antibody negative to hepatitis A, C, and D viruses, and did not have other liver diseases, including alcohol abuse and autoimmune hepatitis. No subjects had decompensated liver disease (evidence or history of ascites, variceal bleeding, or hepatic encephalopathy). The patients were treated with pegylated IFN-α viasubcutaneous injection once a week for 24 weeks. Clinical and laboratory data from the patients were collected at week 0 (before treatment) and at week 24 (after treatment).

### Laboratory assays

#### Plasma HBV DNA monitoring and Serological assessment

Plasma HBV DNA was quantified by TaqMan real-time polymerase chain reaction assay (Roche,USA). The detection sensitivity was 60 copies/mL. HBV DNA (IU/mL) was logarithmically transformed for analysis purposes. Serum levels of liver enzyme alanine aminotransferase (ALT) were measured with a Hitachi-7180 automatic biochemistry analyzer (Hi-tachi Inc., Japan) following standard laboratory methods.

#### Regeants

Staining antibodies CD127-FITC, CD244-FITC, CD3-FITC,CXCR4-PE, PD-1-APC, CD8-PerCP, 7-AAD-PerCP, CD3-APC, and CD4-PerCP were purchased from BD Biosciences (San Jose, CA, USA), CCR7-FITC from eBioscience (San Diego, CA, USA), IFN-γ-FITC, CD45RO-PE from R&D Systems (Minneapolis, MN, USA), CD45RO-APC from BD pharmingen (San Jose, CA, USA), and Granzyme B-FITC and Perforin -PE from BioLegend (San Diego, CA, USA).

#### Blood cell staining

Freshly heparinized blood samples collected from CHB patients were aliquoted into different tubes. Each tube was incubated with the antibodies combination as the study designed and corresponding antibody isotypes. The samples were then processed by a red blood cell lysing solution (BD, CA, USA) and washed twice with phosphate buffered saline (PBS, Gibco, USA)), and fixed with 1% paraformaldehyde. As for ICCS, lysed blood samples were first stained with surface antibodies combination, and then were washed, fixed and permeabilized (Fix/Perm, eBioscience) according to the manufacturer instructions.

In IFN-γ stimulation assay, 200μL fresh whole blood cells from patients were cultured in 800μL RPMI-1640 medium containing 10% fetal calf serum (FCS), PMA (300 ng/ml; Sigma-Aldrich, St. Louis, MO, USA), ionomycin (100 ng/ml Invitrogen, Ontario, Canada), and Golgi Stop (1 μl/ml, BD San Jose, CA, USA) at 37°C, 5% CO_2_ for 5 hours. ICCS was repeated. Cell viability was assessed by 7-aminoactinomycin D (7-AAD) staining (BD Biosciences, San Jose, CA, USA).

#### PBMC isolation and cell stimulation *in vitro*

Freshly heparinized blood samples from CHB patients were two-fold diluted with RPMI-1640 medium containing 10% heat-inactivated FCS. PBMC were isolated by Ficoll-PaqueTM PLUS (GE Healthcare Bio-sciences, Sweden) and resuspended in RPMI-1640 medium supplemented with 300 μg/mL L-glutamin, 100 U/mL penicillin, 100 μg/mL streptomycin, and 10% FCS. The isolated PBMC were then cultured with recombinated HBV core antigen (1 ng/ml, 1–183 amino acid, Cali-Bio, USA) and recombinant human IL-2 (50 IU/ml, Peprotech, UK) in 24-well plates (Corning, USA) at the density of 5 × 10^5^/mLfor 72 hours before they were stimulated withPMA and ionomycin following the procedure described in IFN-γ stimulation assay. The release of intracellular cytokines from Golgi body was blocked by using Golgi Plug (1 μl/ml, BD San Jose, CA, USA) in this assay.

#### Flow cytometry

Flow cytometric analysis was performed on the FACS Calibur 4-color flow cytometer (Becton Dickinson, USA), and immunophenotypic sorting was analyzed using Flow Jo, version 7.5 (Tree star, OR, USA). Staining panels and antibody combinations are shown in the Table 2.

**Table 2:** Combinations of staining antibody panels

	**FITC**	**PE**	**PerCP**	**APC**
Panel 1	CD3	CD45RO	CD4	PD-1
Panel 2	CD3	CD45RO	CD8	PD-1
Panel 3	CD244	CD45RO	CD4	CD3
Panel 4	CD244	CD45RO	CD8	CD3
Panel 5	Granzyme B	CD45RO	CD8	CD3
Panel 6	CD3	Perforin	CD8	CD45RO
Panel 7	CD3	IFN	CD4	CD45RO
Panel 8	CD3	IFN	CD8	CD45RO
Panel 9	CD3	None	7-AAD	CD45RO

Each row represents a combination of four staining antibodies in each panel; each column represents the different antibodies in each flow cytometry channel.

#### Statistical analysis

All data were analyzed using GraphPad Prism 5.0 software (San Diego, CA, USA). Statistical differences between groups were determined by using the nonparametric Mann–Whitney *U* test. Data from the same individuals were compared by using the Wilcoxon matched pairs test. Correlations between variables were evaluated using Spearman method. For all tests, a *P*-value of less than 0.05 was considered to be a significant difference.

## Abbreviations

IFN: Interferon; CHB: Chronic hepatitis B; PD-1: Programmed death-1; PD-L1: Programmed death ligand-1; ICCS: Intracytoplasmic cellular staining; PBMC: Peripheral blood mononuclear cell; PMA: Phorbol 12-myristate-13 acetate; PBS: Phosphate buffered saline; IL: Interleukin.

## Competing interests

The authors declare no financial or commercial competing interests.

## Authors’ contributions

LY performed the laboratory work and drafted the paper. HF was in charge of collecting the clinical samples and analyzing the data and was involved with writing. RY, LS, and DP performed laboratory work. WG designed the project, revised the paper, and supported all work. All authors read and approved the final manuscript.
